# PRSS1 Upregulation Predicts Platinum Resistance in Ovarian Cancer Patients

**DOI:** 10.3389/fcell.2020.618341

**Published:** 2021-01-28

**Authors:** Linan Xing, Songyu Tian, Wanqi Mi, Yongjian Zhang, Yunyan Zhang, Yuxi Zhang, Fengye Xu, Chunlong Zhang, Ge Lou

**Affiliations:** ^1^Department of Gynecology, Harbin Medical University Cancer Hospital, Harbin, China; ^2^College of Bioinformatics Science and Technology, Harbin Medical University, Harbin, China

**Keywords:** PRSS1, ovarian cancer, platinum response, random walk analysis, low-throughput experiment

## Abstract

Ovarian cancer is the most frequent cause of death among gynecologic malignancies. A total of 80% of patients who have completed platinum-based chemotherapy suffer from relapse and develop resistance within 2 years. In the present study, we obtained patients' complete platinum (cisplatin and carboplatin) medication information from The Cancer Genome Atlas database and then divided them into two categories: resistance and sensitivity. Difference analysis was performed to screen differentially expressed genes (DEgenes) related to platinum response. Subsequently, we annotated DEgenes into the protein–protein interaction network as seed nodes and analyzed them by random walk. Finally, second-ranking protease serine 1 gene (PRSS1) was selected as a candidate gene for verification analysis. PRSS1's expression pattern was continuously studied in Oncomine and cBio Cancer Genomic Portal databases, revealing the key roles of PRSS1 in ovarian cancer formation. Hereafter, we conducted in-depth explorations on PRSS1's platinum response to ovarian cancer through tissue and cytological experiments. Quantitative real-time polymerase chain reaction and Western blot assay results indicated that PRSS1 expression levels in platinum-resistant samples (tissue/cell) were significantly higher than in samples sensitive to platinum. By cell transfection assay, we observed that knockdown of PRSS1 reduced the resistance of ovarian cancer cells to cisplatin. Meanwhile, overexpression of PRSS1 increased the resistance to cisplatin. In conclusion, we identified a novel risk gene PRSS1 related to ovarian cancer platinum response and confirmed its key roles using multiple levels of low-throughput experiments, revealing a new treatment strategy based on a novel target factor for overcoming cisplatin resistance in ovarian cancer.

## Introduction

Ovarian cancer is the second most frequent cause of cancer death in women worldwide. Most female patients are diagnosed with stage III/IV ovarian cancer for the first time, and more than 75% of patients die from the disease (Banerjee and Kaye, 2013). Although platinum-based standardized treatment has achieved progress, the responses are often temporary. In total, 80% of patients develop platinum-based resistance and experience relapse (Bookman et al., 2009). The 5-year survival rate is 47% (Lheureux et al., 2019). Therefore, revealing the mechanism of platinum resistance is an urgent requirement for the treatment of ovarian cancer.

Growing evidence has indicated that bioinformatics methods for mining genome data sets can be used to understand the biological mechanisms of platinum resistance in ovarian cancer, screen new biomarkers with clinical significance to guide diagnosis, and evaluate prognosis and treatment. Murakami et al. identified 14 DEgenes in responsive and resistant cases from advanced ovarian cancer biopsy expression dataset in the Gene Expression Omnibus database. A scoring system that applies a single-sample gene set enrichment analysis (GSEA) to predict drug response was generated by using these genes. This scoring system may aid in the development of individualized treatments for ovarian cancer (Murakami et al., 2016). In another study on platinum resistance, the transcriptome of primary FIGO IIIc serous ovarian samples was screened in The Cancer Genome Atlas (TCGA) database. Liu et al. established a prognostic model based on seven genes. After systematic analysis, they indicated that the optimized seven-gene-based model can be used as a valuable marker of the response to platinum-based chemotherapy and a valuable and powerful tool for predicting the survival of FIGO IIIc serous ovarian cancer (Liu et al., 2018). Most of the studies mentioned above focused on the differences between platinum-resistant or -sensitive patients through comparison and excluded low-throughput experiments. Hence, candidate platinum response signature based on global network information must be identified and its performance be verified by low-throughput experiments.

**Figure 1:**
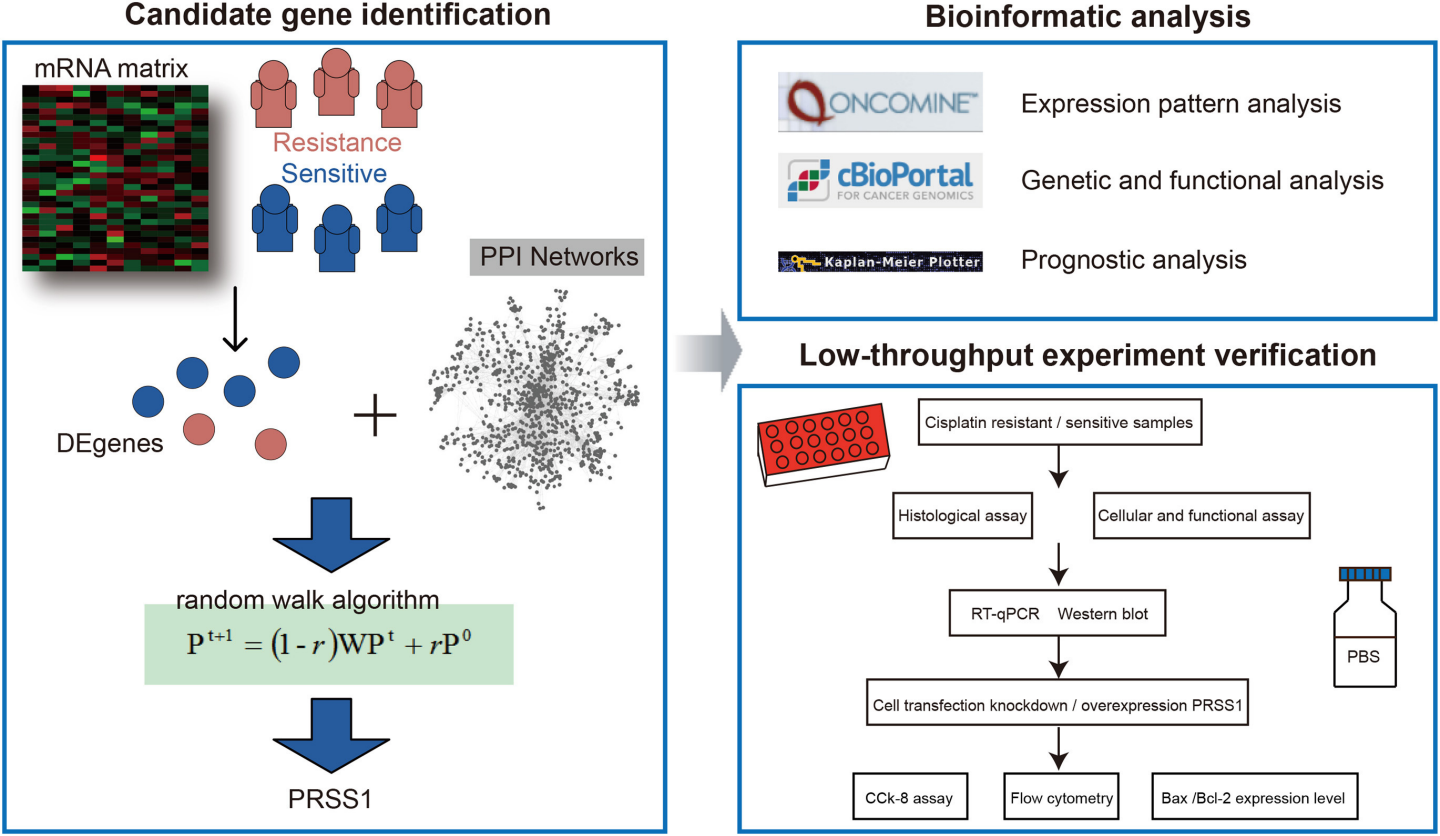
Overall workflow.

In the current study, we first identified DEgenes between platinum-resistant and -sensitive samples from TCGA database and performed global network random walk analysis based on these genes as seeds (Figure 1). After the analysis, we focused on the top-ranked PRSS1 and explored its biological roles in ovarian cancer. Furthermore, the low-throughput verification for this gene was performed in three aspects. First, PRSS1 was verified in platinum-resistant and -sensitive samples, and results showed that PRSS1 was overexpressed in resistant samples and exhibited low expression in sensitive samples. Second, PRSS1 was knocked down to detect alterations in its resistance to platinum. The results showed that knockdown of PRSS1 enhanced the sensitivity of ovarian cancer cells. Finally, the overexpression of PRSS1 weakened the sensitivity of ovarian cancer cells to platinum.

## Materials and Methods

### mRNA Expression Data Sets

TCGA database provides more than 30 different types of cancer and covers the molecular characteristics of more than 20,000 primary cancers (Wang et al., 2016). The mRNA count matrix and patients' clinical information in this study were directly downloaded from TCGA website. Based on clinical medication information, the platinum-sensitive and -resistant samples were further defined as follows: (i) selection of drug information included (cisplatin and carboplatin); (ii) grouping: patients with longer than 6 months of platinum-free treatment interval after the initial treatment were classified into the sensitive group, and those with an interval shorter than 6 months were included in the resistant group. A total of 198 and 100 samples were included in the sensitive and resistant groups, respectively. Supplementary Table 1 provides detailed information on the patients.

### Differential Expression and Random Walk Analyses

The differential expression analysis between platinum-resistant and -sensitive samples was performed using R *edgeR* package. The genes with adjusted *p*-values of < 0.05 were considered DEgenes. Then, we downloaded the protein–protein interaction (PPI) network from STRING database (https://string-db.org/), which is a large-scale database with abundant resources, to establish a comprehensive and objective global network (Szklarczyk et al., 2019). In the current study, an edge threshold >0.8 was considered the final PPI network. The network contained 12,363 protein-coding genes and 559,722 interaction relationships. Based on the obtained DEgenes and PPI network, we further performed a global risk impact analysis (named random walk) to optimize and identify mRNAs. The random walk algorithm was developed for candidate factor analysis; it displays advantages in identifying signature genes on the basis of global network information (Kohler et al., 2008; Zhang et al., 2014; Wang et al., 2020). The related analysis was performed using R *RWOAG* package with default parameters (*r* = 0.6) and equal weight for each DEgene as seed node. Supplementary Table 2 presents the results of random walk analysis.

### Functional Enrichment Analysis

Gene Ontology and Kyoto Encyclopedia of Genes and Genomes (KEGG) enrichment analyses were performed using R *clusterProfiler* package (Yu et al., 2012). In this study, we performed two kinds of enrichment analyses: (i) enrichment analysis of all ranking genes after applying the random walk algorithm using GSEA and (ii) enrichment analysis of PRSS1 co-expression genes with a *p* < 0.05 using the overlapping method.

### Other Online Analyses

To explore PRSS1's biological characteristics, we also utilized other online websites, including those of Oncomine and cBio Cancer Genomic Portal (c-BioPortal).

Oncomine (www.oncomine.org) is a powerful database (Rhodes et al., 2007). In this study, we downloaded and calculated PRSS1's raw expression data on different cancer types. Meanwhile, ovarian cancer and normal tissue expression data from six studies were utilized for analysis (Welsh et al., 2001; Hendrix et al., 2006; Bonome et al., 2008; Yoshihara et al., 2009; Abdallah et al., 2015). The data were statistically drawn by GraphPad Prism 8.3.0. A *P*-value of < 0.05 was considered statistically significant.

In c-BioPortal (http://cbioportal.org), we first selected research on ovarian cancer. Then, PRSS1 co-expression genes were deeply explored. Finally, Cytoscape (version 3.5.1) was used to construct and visualize the functional network of gene co-expression relationships.

### Patients and Specimens

Eight ovarian cancer patients who underwent surgery at the Department of Gynecology, Harbin Medical University Cancer Hospital (Harbin, China) between August 1st, 2018 and December 31st, 2018 were recruited in our study. The inclusion criteria included the following: (i) women diagnosed with ovarian cancer by imaging and pathology; (ii) with complete clinical data; (iii) no previous chemotherapy, radiotherapy, nor immunotherapy before surgery. The exclusion criteria included the following: (i) patients with a history of other cancers; (ii) patients who received chemotherapy, radiotherapy, or immunotherapy before the surgery.

Ovarian cancer patients who received first-line treatment with paclitaxel and platinum after surgery and had disease progression or recurrence within 6 months were defined as platinum-resistant; a patient was defined as platinum-sensitive when no evidence proving disease progression nor recurrence was found 6 months after receiving primary treatment (Gordon et al., 2001). In this study, four patients were classified as platinum-resistant, and four were platinum-sensitive. All tumor tissues were collected and stored in liquid nitrogen for analysis. This study complied with the Helsinki Declaration and was approved by the Ethics Committee of Harbin Medical University (Harbin, China). Informed consent was obtained from all patients or their family members.

### Cell Lines and Culture

SKOV3, A2780DDP, and SKOV3DDP cell lines were cultured in Roswell Park Memorial Institute 1640 (Beijing Labgic Technology Co., Ltd., China), whereas the A2780 cell line was cultured in Dulbecco's Modified Eagle Medium (Beijing Labgic Technology Co., Ltd., China). The SKOV3DDP cell line was added with 0.2 μM cisplatin during the culture process. All cell lines were purchased from Shanghai Chuanqiu Biotechnology Co., Ltd., China. Each kind of medium was supplemented with 10% fetal bovine serum in a humid incubator containing 5% CO_2_ at 37°C.

### Quantitative Real-Time Polymerase Chain Reaction (RT-qPCR) Assay

The PRSS1 mRNA expression in ovarian cancer cell lines and fresh frozen ovarian cancer tissue samples was determined by RT-qPCR. The total RNAs were isolated by TRIpure reagent (BioTeke Corporation, Beijing, China) following the manufacturer's instructions. RNA was synthesized to cDNA using super M-MLV reverse transcriptase (BioTeke Corporation, Beijing, China). qPCR was performed using a StepOnePLusTM Real-Time PCR System (Applied Biosystems, Foster City, CA, USA) and SYBR-Green assay kit (Solarbio, Beijing, China) following the manufacturers' instructions. Supplementary Table 3 shows the primer sequences used in this study. The gene expression was calculated by the 2^−ΔΔCT^ method.

### Western Blot Assay and Antibodies

Western blot assay was performed to determine the expression levels of all proteins (including PRSS1, Bcl-2, and Bax) in tissues and cell lines. The total proteins in cells were collected using radioimmunoprecipitation assay lysis buffer, and the enhanced bicinchoninic acid protein assay kit was used to detect protein concentration. The proteins were then separated by 10% sodium dodecyl sulfate-polyacrylamide gel electrophoresis, and the product was transferred to a polyvinylidene fluoride (PVDF) membrane and finally blocked with 5% skim milk. Subsequently, the PVDF membrane was incubated with a primary antibody overnight, washed with phosphate buffered saline (PBS) with Tween® 20, and incubated with the secondary antibody for 1 h. The antibodies were finally visualized by detection of the bound antibody using ECL chemiluminescence kit. Supplementary Table 4 describes all antibodies used in this study.

### Knockdown and Overexpression Studies

Cells were seeded at a density of 4 × 10^5^ cells/well into six-well plates and incubated until the density was about 70% after cell adherence to the wall of an incubator at 37°C in 5% CO_2_. A2780DDP and Skov3DDP cell lines were transfected with siRNA and the negative control to knock down PRSS1 expression. Supplementary Table 3 displays the sequence of PRSS1-SiRNA (Si-PRSS1). We transfected PRSS1 overexpression plasmid (P-PRSS1) (pcDNA3.1-untagged vector, HindIII/XhoI, Sino Biological) or control empty vector with A2780 and Skov3 cancer cells. The transfection reagent (Lipofectamine 2000, Invitrogen, Carlsbad, USA) was added to a six-well plate in accordance with the manufacturer's instructions. The transfection efficiency of cells was tested by RT-qPCR assay 24 h after transfection and Western blot assay after 48 h.

### Cell Counting Kit-8 (CCK-8) Assay

CCK-8 assay (Dojindo Molecular Technologies, Inc., Rockville, MA, USA) was used to analyze cell viability after different treatments. Briefly, cells were seeded at a density of 3 × 10^3^ cells/well in 96-well plates overnight. After incubation with cisplatin at the indicated doses for 24 h at 37°C and under 5% CO_2_, CCK-8 (10 μl) was added to each well. The optical density of each well was measured with a microplate reader (Bio-Rad Laboratories, Inc., Hercules, CA, USA) at 450 nm after incubation. CCK-8 assay was used to compute the semi-inhibitory concentration (IC50) of cells. GraphPad Prism 8.3.0 was used to accurately estimate the IC50 of cisplatin based on log(inhibitor) vs. response-variable slope (four parameters) equation under the non-linear regression dialog.

### Flow Cytometry for Cell Apoptosis Analysis

The cells were collected by centrifugation, washed with PBS, and centrifuged, whereas the supernatant was discarded. Using an Annexin V-fluorescein isothiocyanate (FITC) apoptosis detection kit (Wan Lei Biological Technology Co., Ltd., Shenyang, China), 195 μl binding buffer was added to the cells, followed by dropwise addition of Annexin V-FITC (5 μl) and propidium iodide (PI) (10 μl). Apoptosis was observed by flow cytometry after 15 min of non-light incubation at room temperature.

## Results

### Screening Candidate Genes Related to Platinum Response in Ovarian Cancer Based on Random Walk Algorithm

**Figure 2:**
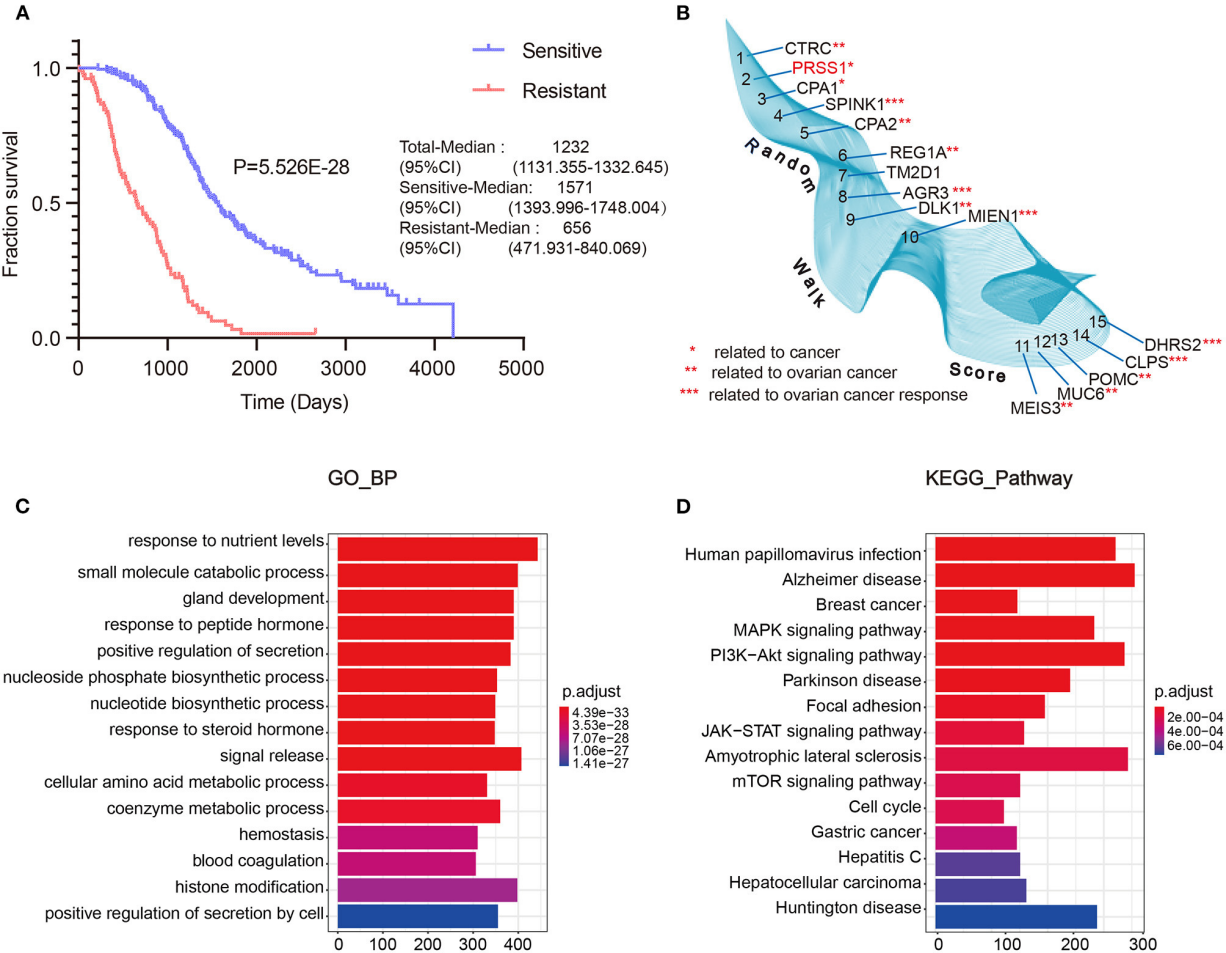
TCGA data integrated with screening and optimization of functional candidate genes. OS analysis of platinum-sensitive/resistant patients **(A)**. Results of differential expression analysis with random walk of platinum-sensitive/resistant patients **(B)**. Enrichment analysis results of BP **(C)** and KEGG pathway **(D)**.

To fully understand the characteristic differences in ovarian cancer platinum response, we compared the clinicopathological factors of platinum-resistant and -sensitive patients. As shown in Table 1, the total number of sensitive patients to all clinical factors was higher than that of resistant patients. Age and response to platinum-based chemotherapy treatment were significantly different between resistant and sensitive patients. The platinum-based chemotherapy response was assessed to evaluate the efficacy of chemotherapy after primary treatment. We further displayed the detailed information of patients, including complete response (CR), partial response (PR), progressive disease, and stable disease, from the two groups (Eisenhauer et al., 2009). In response to platinum-based chemotherapy, the *P*-value of the two groups was 4.0E-6. After completing the primary platinum-based chemotherapy, patients in the CR and PR groups accounted for 87.7% of the total. Among these groups, 26.9% of patients exhibited good results but developed platinum resistance within 6 months. By analyzing the patients' overall survival (OS), those who were sensitive to platinum had a better OS than platinum-resistant ones (Figure 2A).

**Table 1:** Clinical factors of platinum-sensitive/resistant patients' statistical analysis.

**Characteristics**	**Total (*n*, %)**	**Sensitive (*n*, %)**	**Resistant (*n*, %)**	***P*-value**
**Age**	298	198 (66.4%)	100 (33.6%)	1.0E-2
≤ 59	165 (55.4%)	120 (40.3%)	45 (15.1%)	
>59	133 (44.6%)	78 (26.2%)	55 (18.5%)	
**Stage**	298	198 (66.4%)	100 (33.6%)	2.6E-1
I+II	13 (4.4%)	11 (3.7%)	2 (0.7%)	
III+IV	285 (95.6%)	187 (62.8%)	98 (32.9%)	
**Grade**	293	194 (66.2%)	99 (33.8%)	3.0E-1
1 + 2	38 (13.0%)	28 (9.6%)	10 (3.4%)	
3 + 4	255 (87.0%)	166 (56.7%)	89 (30.4%)	
**Chemotherapy response**	212	135 (63.7%)	77 (36.3%)	4.0E-6
CR+PR	186 (87.7%)	129 (60.8%)	57 (26.9%)	
PD+SD	26 (12.3%)	6 (2.8%)	20 (9.4%)	

Next, we performed differential expression analysis on the resistant/sensitive patients. All DEgenes were applied as seed nodes and annotated into the integrated PPI network. Random walk analysis was performed to optimize candidate genes. Figure 2B shows that SPINK1 (4th) (Fioretti et al., 1992), AGR3 (8th) (Fioretti et al., 1992), MIEN1 (10th) (Leung et al., 2013), CLPS (14th) (Han et al., 2012), and DHRS2 (15th) (Song et al., 2009) are relevant to the studies of ovarian cancer platinum response.

**Figure 3:**
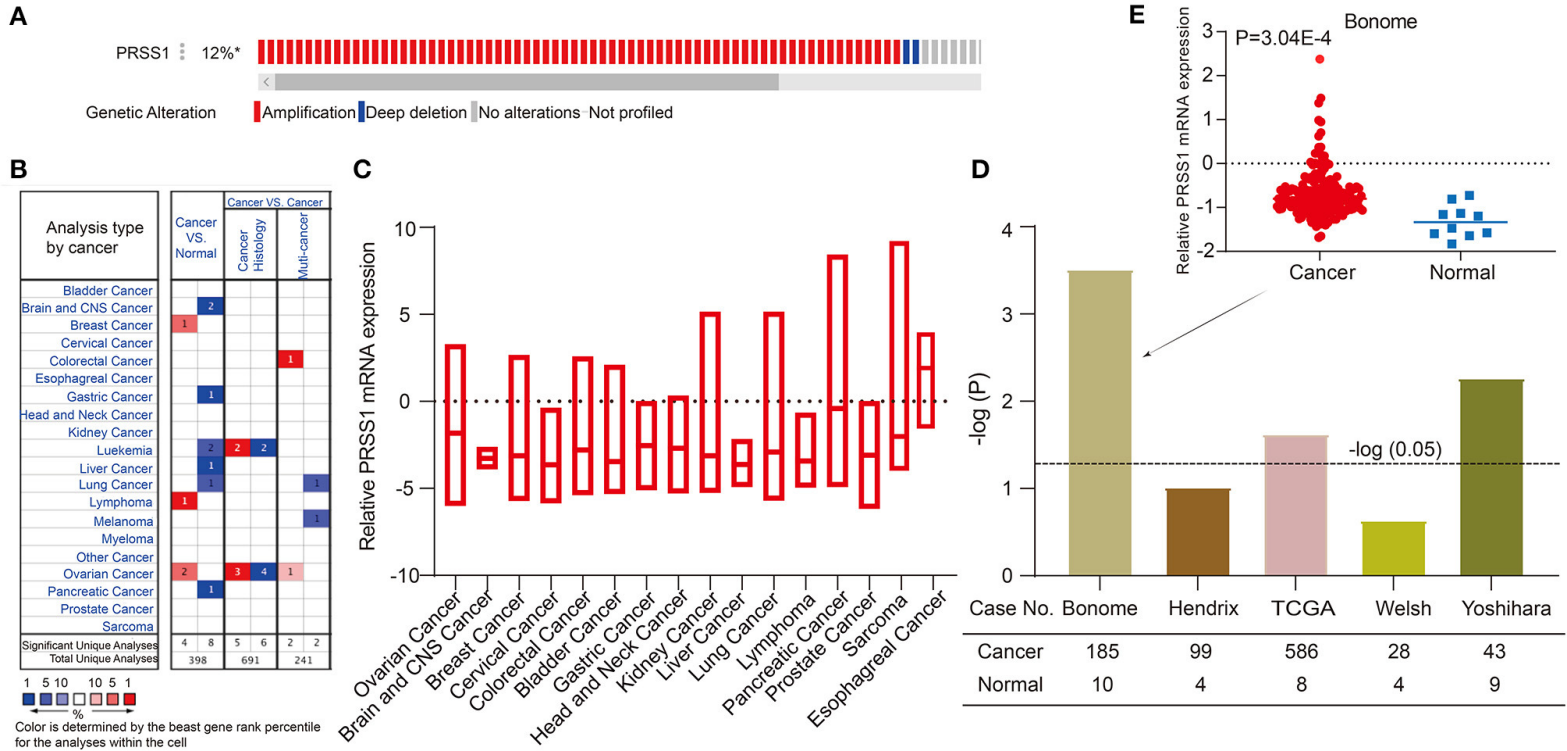
Comprehensive analysis and verification of PRSS1. PRSS1 genetic alterations **(A)**. Analysis of PRSS1 expression in different cancers in Oncome database **(B)**. PRSS1 expression analysis in multiple cancer tissues **(C)**. Significant expression comparison of PRSS1 in ovarian cancer and normal tissues **(D)**. PRSS1 expression in ovarian cancer and normal tissues in Benome database **(E)**.

Furthermore, we performed functional enrichment analysis of biological process (BP) and KEGG on the outcomes of random walk analysis. Figure 2C exhibits the BP results, showing that genes were all enriched in small-molecule catabolic process and response to peptide hormone. As shown in Figure 2D, the KEGG results revealed various significant biological pathways, such as breast cancer, MAPK signaling pathway, and PI3K-Akt signaling pathway. The results of these enrichment analyses are related to platinum resistance in ovarian cancer (Wang et al., 2013; Amini-Farsani et al., 2018; Chen et al., 2018). Therefore, our results based on database calculations have a certain degree of reliability, and the top-ranked genes are closely related to platinum response to ovarian cancer. As shown in Figure 2B, the top-ranked gene CTRC has a certain correlation with ovarian cancer. However, as verified by Oncomine database, no difference was observed in the expression of CTRC in ovarian cancer tissues (Supplementary Figure 2). Therefore, we ranked the second candidate gene PRSS1. This gene has not been confirmed in ovarian cancer research, which adds innovation to our research. Through verification in Oncomine database, we confirmed the significantly different PRSS1 expression in ovarian cancer tissues (Figure 3). Therefore, this gene was selected for subsequent verification analysis.

### PRSS1 Expression Pattern in Multiple Cancers and Co-expression Analysis

In the following research, we validated and analyzed PRSS1 in Oncomine and c-BioPortal databases. Figure 3A shows the schematic of PRSS1 genetic alteration. Two main genetic alterations, namely, amplification and deep deletion, were observed. The frequency of PRSS1 amplification in ovarian cancer is 12%. Figure 3B shows that PRSS1 is over-expressed in breast cancer, lymphoma, and ovarian cancers in the comparison between cancer and normal tissues. Meanwhile, PRSS1 exhibits a low expression in lung, liver, pancreatic, and gastric cancer tissues. Figure 3C shows the PRSS1 expression in various cancer tissues. The lowest expression was detected in liver cancer and the highest in esophageal cancer. Pancreatic cancer had the second-highest expression and ovarian cancer the third. We conducted an in-depth research exploration of ovarian cancer in the Oncomine database. Five studies were based on the expression of PRSS1 in patients with ovarian cancer and normal tissues (see section Materials and Methods). From Figure 3D, PRSS1 was overexpressed in ovarian cancer tissues compared with the normal tissue. PRSS1 presented a significant *P*-value in the Bonome, TCGA, and Yoshihara studies. In the Bonome study, 185 and 10 cancer and normal tissue samples were tested, respectively. The difference was the largest at the *P*-value of 3.04E-4 (Figure 3E).

**Figure 4:**
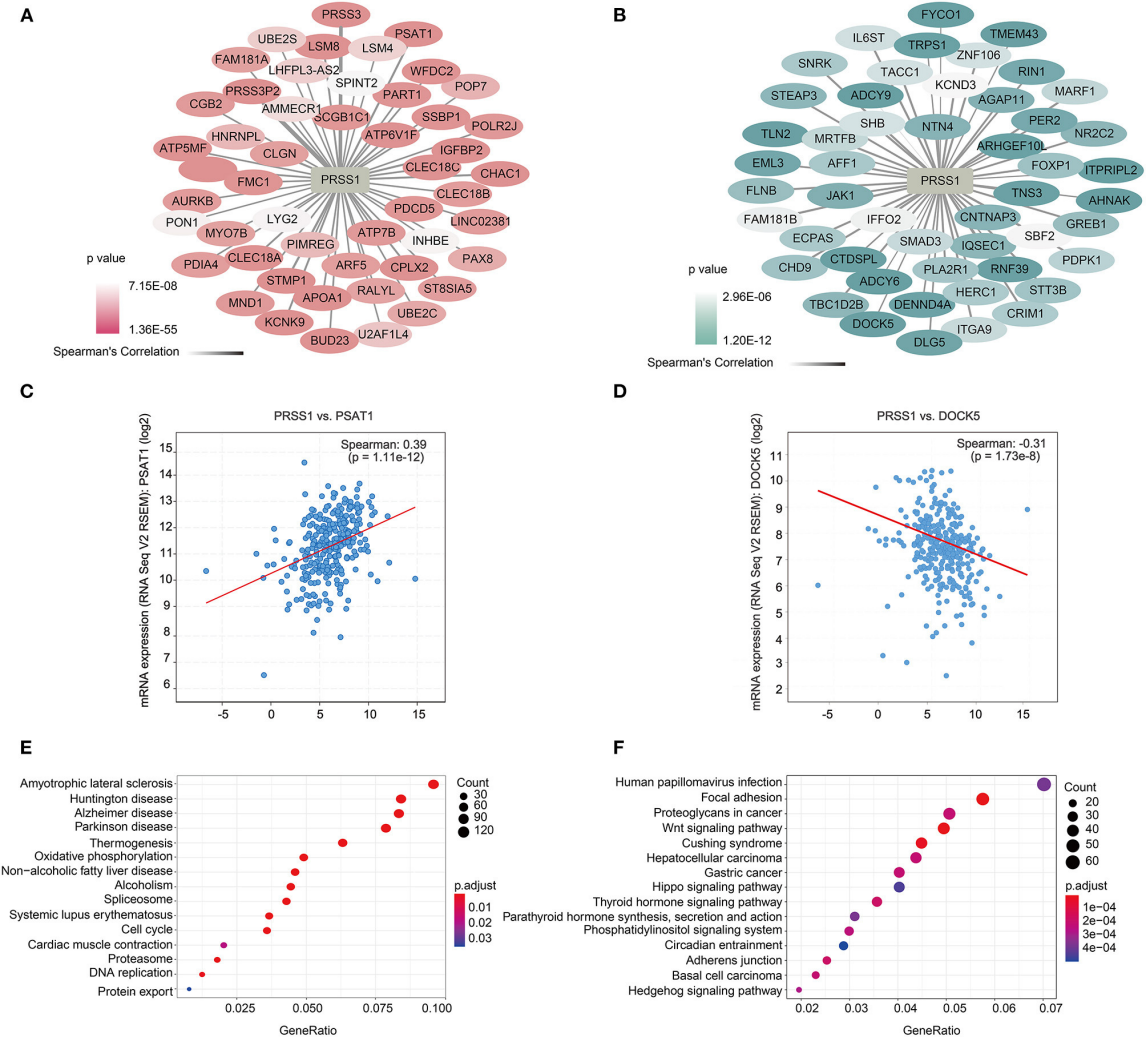
Functional analysis of PRSS1 co-expression genes in c-BioPortal database. Positive **(A)** and negative **(B)** correlation with PRSS1 co-expression and ranking in the top 50 genes. Genes that are positively (and ranked 5th) **(C)** and negatively correlated (and ranked 7th) with PRSS1 co-expression **(D)**. KEGG enrichment analysis of genes that positively **(E)** and negatively **(F)** correlated with PRSS1 co-expression.

To further explore the biological functions of PRSS1, we explored its co-expression genes from c-BioPortal database. A total of 3,210 genes were positively correlated with PRSS1 expression and had a *P* < 0.05 (Figure 4A), whereas 2,241 genes showed a negative correlation (Figure 4B). In the ranking list of positive co-expression genes, the fifth gene PSAT1 was closely related to platinum resistance in ovarian cancer PSAT1 downregulation reduced the resistance and enhanced the sensitivity of ovarian cancer cells to cisplatin (Figure 4C) (Dai et al., 2019). Among all negatively correlated co-expression genes, ANHAK (6th) (Chatterjee et al., 2006) (Sheets et al., 2016) and DOCK5 (7th) exhibited correlations with ovarian cancer (Supplementary Table 3; Figure 4D). Wu et al. pointed out that DOCK5 is down-regulated in ovarian cancer tissues and related to immune response (Wu et al., 2020).

BP and KEGG enrichment analysis results were also assessed by GSEA function for positively/negatively related PRSS1 co-expression genes. The BP results show that positively related genes are mainly involved in the nucleoside triphosphate metabolic process (Supplementary Figure 1A). Maurmann et al. (2015) reported that the concentration of nucleoside triphosphate is related to platinum response in ovarian cancer. The negatively related genes are mainly involved in the Wnt signaling pathway (Supplementary Figure 1B) and significantly enriched in the KEGG results (Figure 4F). Wnt signaling pathway, as confirmed in multiple studies, can regulate platinum response in ovarian cancer in different aspects (Nagaraj et al., 2015; Gu et al., 2019). The results related to platinum resistance in ovarian cancer can also be observed in the KEGG enrichment analysis of positively related genes (Figure 4E) (Dar et al., 2017).

### PRSS1 Was Overexpressed in Platinum-Resistant Patients With Ovarian Cancer

**Figure 5:**
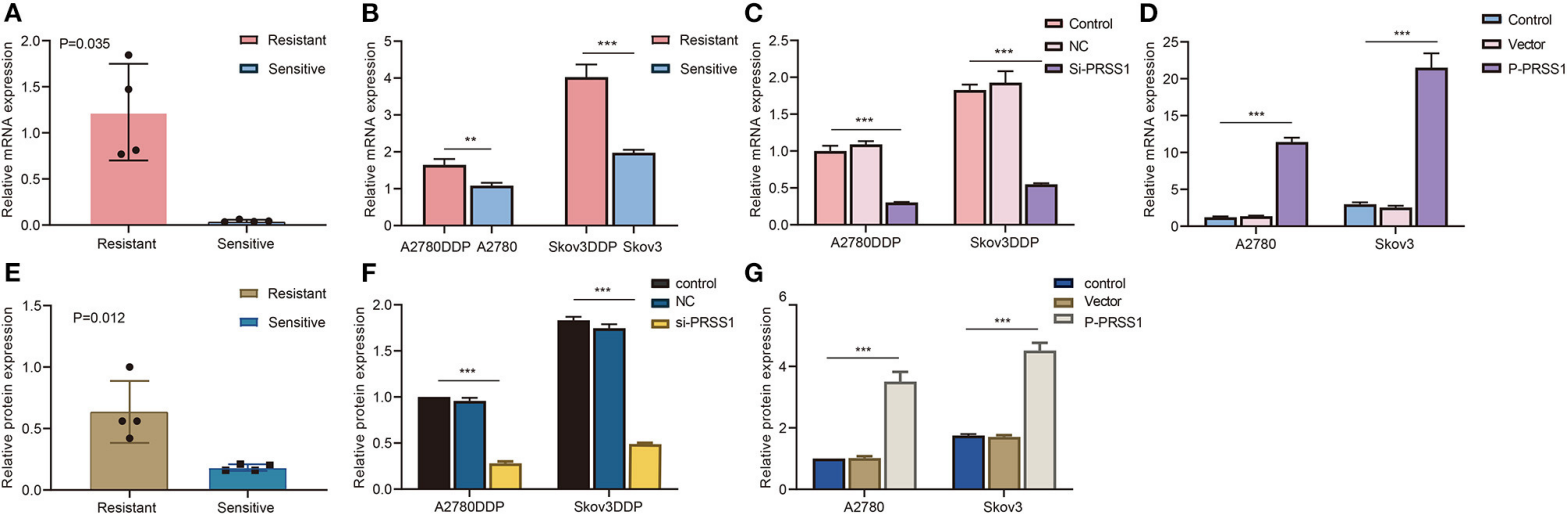
PRSS1 was overexpressed in platinum-resistant samples and transfection assay. RT-qPCR was used to detect the increased mRNA levels of PRSS1 in platinum-resistant ovarian cancer tissues **(A)**. PRSS1 mRNA expression of cisplatin-resistant/sensitive ovarian cancer cells was detected by RT-qPCR **(B)**. Transfection of cisplatin-resistant ovarian cancer cells with SiRNA and application of RT-qPCR assay to detect PRSS1 transfection efficiency after 24 h **(C)**. RT-qPCR was used to detect PRSS1 mRNA expression level in cisplatin-sensitive ovarian cancer cells (A2780 and Skov3) transfected with plasmids **(D)**. Western blot assays demonstrated the increased protein expression of PRSS1 in platinum-resistant ovarian cancer tissues **(E)**. Western blot assays were used to detect the PRSS1 transfection efficiency in cisplatin-resistant ovarian cancer cells after 48 h **(F,G)**. The data are presented as mean ± standard deviation (SD); *n* = 3. **P < 0.01; ***P < 0.001.

To investigate whether PRSS1 is associated with platinum response in ovarian cancer, we detected its differential expression in cisplatin-resistant/sensitive patients. PRSS1 mRNA expression level was examined by RT-qPCR, and the results confirmed its high expression in cancer tissues of cisplatin-resistant patients and low expression in cisplatin-sensitive patients, with a *p*-value of 0.035 (Figure 5A). Western blot analysis was used to detect protein expression, revealing that PRSS1 expression in cancer tissues from cisplatin-resistant patients was significantly higher than that in cisplatin-sensitive patients, with a *P*-value of 0.012 (Figure 5E). The protein expression in ovarian cancer tissues from group A (resistant) patients was significantly higher than that from group B (sensitive) patients (Supplementary Figure 3A).

The mechanism of platinum resistance is relevant to abnormal apoptosis in ovarian cancer. The ratio of Bax (apoptotic protein) and Bcl-2 (anti-apoptotic protein) can precisely regulate tumor cell apoptosis (Burguillos et al., 2011). Its increase can initiate tumor cell apoptosis and enhance the sensitivity to platinum (Reed, 2006). Therefore, we tested the expression levels of Bcl-2 and Bax in the ovarian cancer tissues from cisplatin-resistant/sensitive patients by Western blot assay and calculated the ratio of Bax/Bcl-2. From the results in Supplementary Figure 4A, Bcl-2 protein was overexpressed in the tissues of cisplatin-resistant patients, that is, the expression level in group A (resistant) patients was significantly higher than that in group B (sensitive). The *P*-value of Bcl-2 protein between the two groups was 0.013 (Supplementary Figure 4C). Bax showed a notably higher protein expression level in ovarian cancer tissue from sensitive patients. Supplementary Figure 4B reveals that the protein expression level of group A (resistant) was significantly lower than that of group B (sensitive), with a *P*-value of 0.026 (Supplementary Figure 4D).

### PRSS1 Was Overexpressed in Cisplatin-Resistant Ovarian Cancer Cells

To further study the biological function of PRSS1 in ovarian cancer, we conducted *in vitro* cytology tests on cisplatin-resistant cell lines A2780DDP and Skov3DDP and sensitive cell lines A2780 and Skov3. As presented in Figure 5B, PRSS1 mRNA was highly expressed in cisplatin-resistant cancer cells (A2780DDP and Skov3DDP) compared with sensitive cancer cells (A2780 and Skov3), and the *P*-values were all significant. The expression of Bcl-2 mRNA was consistent with PRSS1 expression. The mRNA expression level of Bcl-2 was up-regulated in cisplatin-resistant cancer cells, but its *P*-value, which was < 0.05, was only significant in the differential expression of A2780DDP and A2780 cells (Supplementary Figure 6A). Contrary to the expression of Bcl-2 mRNA, Bax exhibited low expression in cisplatin-resistant cancer cells, and the *P*-values were significant at < 0.001 (Supplementary Figure 6B).

### Knockdown of PRSS1 Promoted Ovarian Cancer Cells Apoptosis

In terms of previous test results, PRSS1 was overexpressed in cisplatin-resistant cancer cells. Therefore, PRSS1 knockdown increased the sensitivity of cisplatin-resistant cancer cells. PRSS1 was silenced by siRNA, and the cisplatin-resistant cancer cells were tested for mRNA by RT-qPCR assay 24 h after transfection (Supplementary Figure 5A). The protein was tested by Western blot assay after 48 h (Supplementary Figure 5B). As shown in Figure 5C, in cisplatin-resistant cancer cells, the mRNA expression of PRSS1 knockdown group was considerably lower than that of the normal control group, and the *P*-value was < 0.001. As displayed in Supplementary Figure 6C, the mRNA expression of Bcl-2 in the PRSS1 knockdown group was significantly lower than that in the control, and the difference was significant at a *P* < 0.001. Bax mRNA expression was remarkably opposite that of Bcl-2 (Supplementary Figure 6D). The expression of Bax in the PRSS1 knockdown group was significantly higher than that in the control, and the difference was significantly < 0.001. The expressions of PRSS1 and Bcl-2 were positively correlated, whereas Bax expression showed a negative correlation. Therefore, knockdown of PRSS1 expression led to an increased ratio of Bax/Bcl-2, which promoted the apoptosis of ovarian cancer cells (Reed, 2006). Subsequently, we tested the expression of the three genes (PRSS1, Bcl-2, and Bax) by Western blotting. As shown in Supplementary Figure 3B, after PRSS1 knockdown, the PRSS1 protein expression in A2780DDP and Skov3DDP cells was significantly reduced compared with that in the control group, with a *P* < 0.001 (Figure 5F). The same results were verified by experiments at the mRNA level. After knocking down PRSS1, the expression of Bcl-2 at the protein level was significantly reduced (Supplementary Figure 6E), whereas the protein expression level of Bax increased (Supplementary Figure 6F).

### Overexpression of PRSS1 Inhibited Ovarian Cancer Cell Apoptosis

Given that PRSS1 exhibited high expression in cisplatin-resistant cell lines, A2780 and Skov3 cancer cell lines were constructed to overexpress PRSS1 by transfecting them with a plasmid to further explore the platinum-resistant properties of PRSS1. After transfection for 24 h, PRSS1 was successfully overexpressed at the mRNA level as shown by RT-qPCR (Figure 5D), and after 48 h, we determined the protein level by Western blot (Supplementary Figure 3C). The data showed that the protein expression of PRSS1 in A2780 and Skov3 cancer cells was higher than that in the normal control group, and the *P*-value was < 0.001 (Figure 5G). Given the over-expression of PRSS1 in the constructed cancer cell lines, we examined the expression alterations in Bcl-2 and Bax at the mRNA and protein levels. Supplementary Figure 7A shows that Bcl-2 expression was significantly higher than that of the control as PRSS1 mRNA expression increased, and the difference was significant with a *P*-value of < 0.001. Consistent with the results of previous studies, Bax showed a negative correlation with the expressions of PRSS1 and Bcl-2. Given the PRSS1 overexpression, the mRNA expression level of Bax decreased (Supplementary Figure 7B). The protein expression levels of Bcl-2 and Bax were the same as the alterations in mRNA expression levels. Supplementary Figure 7C reveals that after PRSS1 overexpression, Bcl-2's protein expression in the cell line increased. The gray value of Bcl-2 in Skov3 cancer cells was deeper than that of A2780. The protein expressions of Bax and Bcl-2 exhibited opposite trends. In A2780 cancer cells, the expression level of Bax protein in the PRSS1 overexpression group was lower than that in the control group, and the *P*-value was significantly < 0.001 (Supplementary Figure 7D). Thence, PRSS1 overexpression caused the decrease in Bax/Bcl-2 ratio, which halted the apoptosis of ovarian cancer cells.

### Knockdown of PRSS1 Reversed Platinum Resistance, and Its Overexpression Attenuated Sensitivity

**Figure 6:**
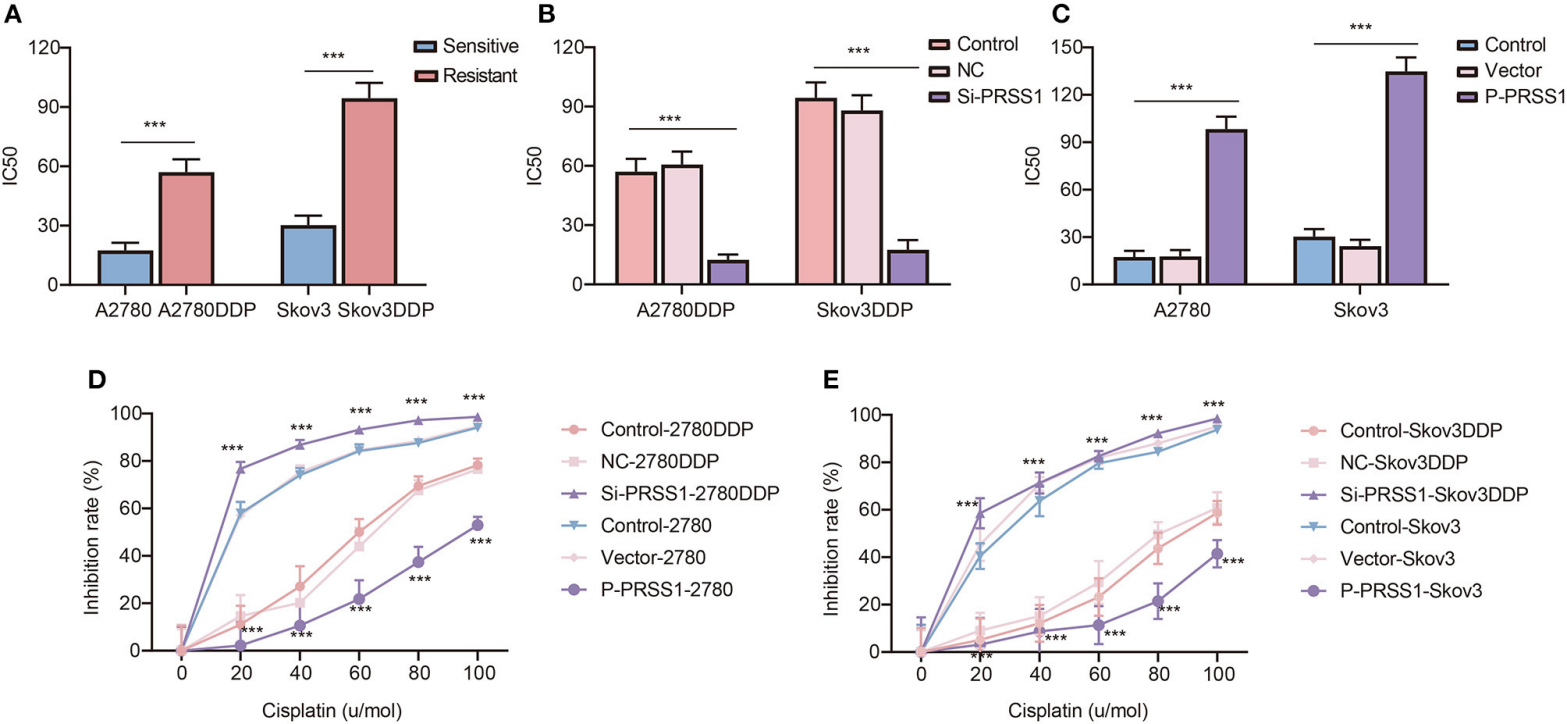
Knockdown of PRSS1 reversed the resistance of ovarian cancer cells to platinum, whereas PRSS1 overexpression reduced their sensitivity. IC50 values of cisplatin-resistant/sensitive ovarian cancer cells exposed to cisplatin **(A)**. After downregulating **(B)** or up-regulating **(C)** PRSS1, the IC50 value of ovarian cancer cells exposed to cisplatin was calculated by using GraphPad 8.3.0 software. Following the treatment of cells with different concentrations of cisplatin, CCK-8 assay was used to detect cell viability, and the inhibition rate was calculated **(D,E)**. The data are presented as the mean ± SD; *n* = 3. ***P < 0.001.

By applying transfection-constructed cisplatin-resistant and sensitive cells, we performed cellular biological function assays on PRSS1 to study its platinum response mechanism. CCK-8 assay was used to calculate the IC50 values and measure the cell inhibition rate. Figure 6A manifests that the IC50 value of cisplatin-resistant cancer cell lines was significantly higher than that of sensitive cells, and the *P*-value of the two groups was statistically significant at < 0.001. After knocking down the expression of PRSS1 in cisplatin-resistant cancer cell lines, the IC50 value in the cell lines was significantly reduced compared with the control (Figure 6B). However, when PRSS1 was overexpressed, the IC50 value of sensitive cancer cell lines increased significantly (Figure 6C). Subsequently, all cancer cells were tested by different concentrations of cisplatin (20, 40, 60, 80, and 100 μ/mol). The results showed that the growth inhibition rate of all cancer cell lines increased with the increase in cisplatin concentration. The results in Figures 6D,E prove that the growth inhibition rate was the highest after the knockdown of PRSS1 expression, whereas the growth inhibition rate was the lowest after overexpression of PRSS1, respectively. CCK-8 assay indicated that the cell lines with low PRSS1 expression increased the growth inhibition rate with the gradual rise in cisplatin concentration. Nevertheless, the growth inhibition rate decreased.

Annexin V-FITC assay was employed to determine apoptosis in all cancer cells. The characteristics shown in Supplementary Figure 8 indicate that by knocking down the PRSS1 expression in A2780DDP cells, the number of early and late apoptotic cancer cells increased to varying degrees. However, as observed in A2780 cells, the total number of apoptotic cancer cells decreased due to the increased expression of PRSS1. From the above results, we can conclude that knockdown of PRSS1 can enhance the sensitivity of cisplatin-resistant cancer cell lines, whereas its overexpression can weaken the sensitivity of cisplatin-sensitive cancer cell lines.

## Discussion

Among gynecological malignancies, ovarian cancer is the leading cause of death in affected women worldwide. The care standard relies on platinum treatment for patients with ovarian cancer. However, the chemoresistance of ovarian cancer is the main challenge to improve prognosis (Cui et al., 2018). Therefore, elucidating the tumor's molecular mechanism will help in optimizing platinum-based chemosensitization strategies. In the present study, based on the bioinformatics strategy of optimizing DEgenes by applying TCGA data, PRSS1 was screened as a functional candidate gene for platinum response research. Then, we conducted expression analysis and verification of PRSS1 in large databases and performed a series of low-throughput experiments to explore the platinum response mechanism of PRSS1. As verified by scientific low-throughput test, PRSS1 can be a potential biomarker for predicting platinum response in ovarian cancer.

PRSS1 encodes human cationic trypsinogen 1 (Sahin-Tóth, 2005; Salameh and Radisky, 2013), which can be synthesized in pancreatic acinar cells and secreted into the small intestine to promote digestive function (Wang et al., 2019). Mutations and abnormal regulation of PRSS1 play important roles in the occurrence and development of malignant tumors (Song et al., 2012; Ludovini et al., 2016; Erinjeri et al., 2018). PRSS1 mutations can increase the risk of pancreatic cancer and are closely related to susceptibility to gastric cancer (Chen et al., 2017; Na et al., 2020). However, their role in promoting ovarian cancer mechanism and ovarian cancer platinum resistance remains to be addressed. In Oncomine database, PRSS1 was more highly expressed in ovarian cancer tissues than in normal tissues. Its expression in ovarian cancer ranked the top in the multiple-cancer expression analysis (Figure 3). In the results of enrichment analysis, the pathway involving PRSS1 and its co-expression genes was closely related to platinum resistance in ovarian cancer. The findings indicate that PRSS1 and its negatively related genes are involved in the Wnt signaling pathway (Figure 4F). The abnormal activation of the Wnt signaling pathway is significant in the development, invasion, metastasis, and platinum resistance of ovarian cancer (Arend et al., 2013; Gu et al., 2019; Li et al., 2019; Yen et al., 2019). A study has shown that miR-1180 activates the Wnt signaling pathway in cancer cells. Wnt signal increases the level of glycolysis, thereby enhancing the chemoresistance of ovarian cancer cells (Gu et al., 2019). This event was detected from our analysis results, that is, PRSS1 and its positively related genes were involved in mitochondrial gene expression (Supplementary Figure 1A). Mitochondria are mainly involved in ATP production, and their normal function depends on the crosstalk between mitochondrial and nuclear genomes. The dysfunction of mitochondria expression may cause disease in humans (Kotrys and Szczesny, 2019). The expression of mitochondrial genes is also correlated with platinum resistance in ovarian cancer. The expression of mitochondrial gene RNR1 might be used as a predictor of tumor sensitivity to platinum chemotherapy (Bragoszewski et al., 2008).

Resistance is the most serious disadvantage of platinum-based chemotherapy. The related molecular mechanism must be clarified to improve chemotherapy sensitivity and reverse transformation resistance. In our study, we manifested that PRSS1 was overexpressed in platinum-resistant cancer tissues/cells and lowly expressed in platinum-sensitive ones. Moreover, knockdown of PRSS1 increased platinum sensitivity, and overexpression reduced platinum resistance in ovarian cancer. Platinum's main target is DNA, and triggering DNA damage is the basis for patients' sensitivity to platinum. If the ability to repair DNA damage is abnormally increased, cells will be able to repair the damage caused by chemotherapy agents, eventually leading to chemotherapy resistance (Teng et al., 2014). Therefore, abnormal PRSS1 mutations trigger the amplification of tumor gene fragments and increase the abnormal expression of tumor-related proteins, thereby improving the ability to repair DNA damage. Over time, ovarian cancer patients develop resistance to platinum-based chemotherapy.

We combined the high-throughput data with low-throughput experiments to identify and verify the vital roles of the novel marker PRSS1. Cancer tissues/cells of platinum-resistant/sensitive patients were used to verify PRSS1 at the mRNA and protein expression levels. After a series of platinum response cell function assays, the role of PRSS1 in platinum response mechanism has been confirmed. However, other biological roles that are related to platinum response of PRSS1 in ovarian cancer, including invasion, angiogenesis, epithelial-mesenchymal transition, and cell survival, need to be further investigated. The current study can provide a novel framework for the study of platinum response in ovarian cancer and identify a new platinum resistance gene for potential mechanism research or clinical use.

## Data Availability Statement

The raw data supporting the conclusions of this article will be made available by the authors, without undue reservation.

## Ethics Statement

The studies involving human participants were reviewed and approved by Harbin Medical University. The patients/participants provided their written informed consent to participate in this study. Written informed consent was obtained from the individual(s) for the publication of any potentially identifiable images or data included in this article.

## Author Contributions

GL, CZ, and LX: conception and design. ST, LX, YonZ, and FX: acquisition of data. CZ, LX, WM, YonZ, and YuxZ: analysis and interpretation of data. LX and CZ: writing the manuscript. GL, CZ, and YunZ: study supervision. All authors: read and approved the final manuscript.

## Conflict of Interest

The authors declare that the research was conducted in the absence of any commercial or financial relationships that could be construed as a potential conflict of interest.

## Supplementary Material

Supplementary Figure 1 BP enrichment analysis of genes that are positively **(A)** and negatively **(B)** correlated with PRSS1 co-expression.

Supplementary Figure 2 Analysis of CTRC expression in different cancers in Oncome database.

Supplementary Figure 3 Western blot images of PRSS1. PRSS1 was highly expressed in platinum-resistant ovarian cancer tissues **(A)**. Group A, ovarian cancer tissues of platinum-resistant patients; Group B, ovarian cancer tissues of platinum-sensitive patients. Detection of transfection efficiency after the knockdown of PRSS1 in A2780DDP and Skov3DDP cells for 48 h **(B)**. Transfection of A2780 and Skov3 cells with plasmid to overexpress PRSS1; Western blot assay was used to detect PRSS1 transfection efficiency after 48 h **(C)**. All experiments were repeated thrice.

Supplementary Figure 4 Bcl-2 and Bax up-regulated in platinum-resistant and platinum-sensitive tissues, respectively. Western blot assays demonstrated the protein expression of Bcl-2 **(A,C)** and Bax **(B,D)** in platinum-resistant/sensitive ovarian cancer tissues. Group A, ovarian cancer tissues of platinum-resistant patients; Group B, ovarian cancer tissues of platinum sensitive patients. The data were presented as the mean ± SD; *n* = 3.

Supplementary Figure 5 Knocked down PRSS1 expression in platinum-resistant cells. RT-qPCR was used to verify the transfection effect of the three Si-PRSS1 24 h after transfection **(A)**. Western blot was applied to detect the transfection effect of the three Si-PRSS1 at 48 h after transfection **(B)**. The data are presented as the mean ± SD; *n* = 3. ^***^*P* < 0.001.

Supplementary Figure 6 Knock down of PRSS1 expression led to an increased ratio of Bax/Bcl-2. Bcl-2 **(A)** and Bax **(B)** mRNA expressions of cisplatin-resistant/sensitive ovarian cancer cells were detected by RT-qPCR. Detection of Bcl-2 **(C)** and Bax **(D)** mRNA expressions by RT-qPCR assay after knockdown of PRSS1. Western blot assays were used to detect alterations in Bcl-2 **(E)** and Bax **(F)** protein expression after PRSS1 downregulation. The data are presented as the mean ± SD; *n* = 3. ^*^*P* < 0.05; ^***^*P* < 0.001.

Supplementary Figure 7 Overexpression of PRSS1 decreased the ratio of Bax/Bcl-2. After upregulation of PRSS1 expression, RT-qPCR was used to detect Bcl-2 **(A)** and Bax **(B)** mRNA expression levels. Western blot assays were used to detect Bcl-2 **(C)** and Bax **(D)** protein expression levels. The data are presented as the mean ± SD; *n* = 3. ^**^*P* < 0.01; ^***^*P* < 0.001.

Supplementary Figure 8 Flow cytometry assay of cell apoptosis in ovarian cancer cells. Early apoptotic population in the Q4 gate was characterized with Annexin V (+) and PI (–), and the late apoptotic population in the Q2 gate was characterized with Annexin V (+) and PI (+). All experiments were repeated thrice.

Supplementary Table 1 Detailed medication and clinical information in TCGA database of platinum-sensitive/resistant patients.

Supplementary Table 2 Results of random walks.

Supplementary Table 3 Results of primer sequences.

Supplementary Table 4 Protein antibodies used for Western blot.

## Abbreviations

PRSS1: The protease serine 1 gene
GSEA: gene set enrichment analysis
Degenes: differentially expressed genes
PPI: protein-protein interaction
TCGA: The Cancer Genome Atlas
KEGG: Kyoto Encyclopedia of Genes and Genomes
c-BioPortal: The cBio Cancer Genomics Portal
OS: overall survival
RT-qPCR: Quantitative real-time polymerase chain reaction
Si-PRSS1: PRSS1-SiRNA
P-PRSS1: PRSS1 overexpression plasmid
PVDF: polyvinylidene fluoride-fluoride
PBS: phosphate buffered saline
FITC: fluorescein isothiocyanate
PI: propidium iodide
OD: Optical density
IC50: semi-inhibitory concentration
CCK-8: Cell Counting Kit-8
CR: complete response
PR: partial response
BP: biological process
SD: standard deviation.
