# Supplementary figures and images for: PRSS1 Upregulation Predicts Platinum Resistance in Ovarian Cancer Patients
Source: Front Cell Dev Biol. 2021 Jan 28;8:618341. doi: 10.3389/fcell.2020.618341 (PMC7876278; doi:10.3389/fcell.2020.618341)

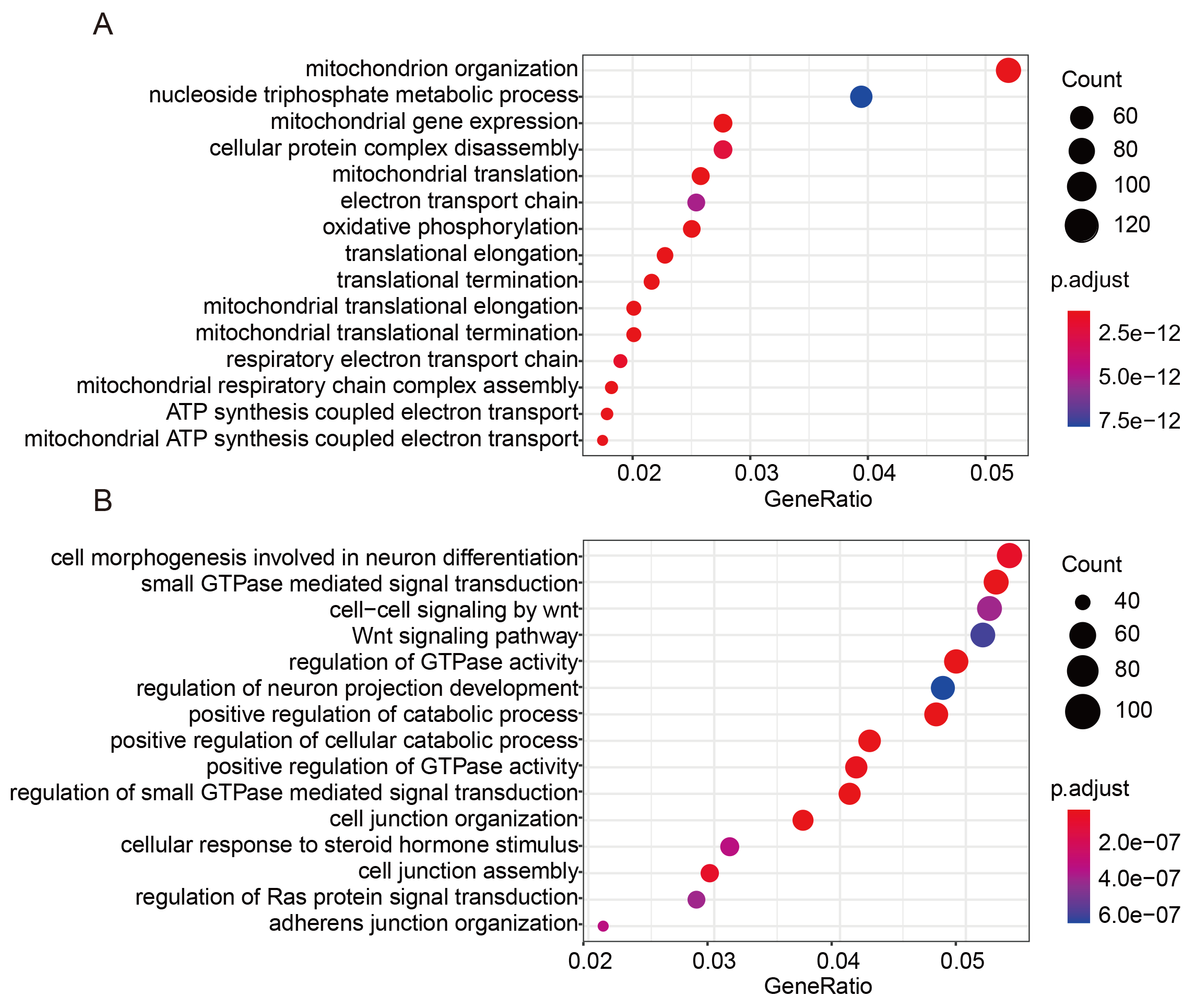

Supplement: Supplementary Figure 1 — BP enrichment analysis of genes that are positively (A) and negatively (B) correlated with PRSS1 co-expression. [file Image_1.TIF]

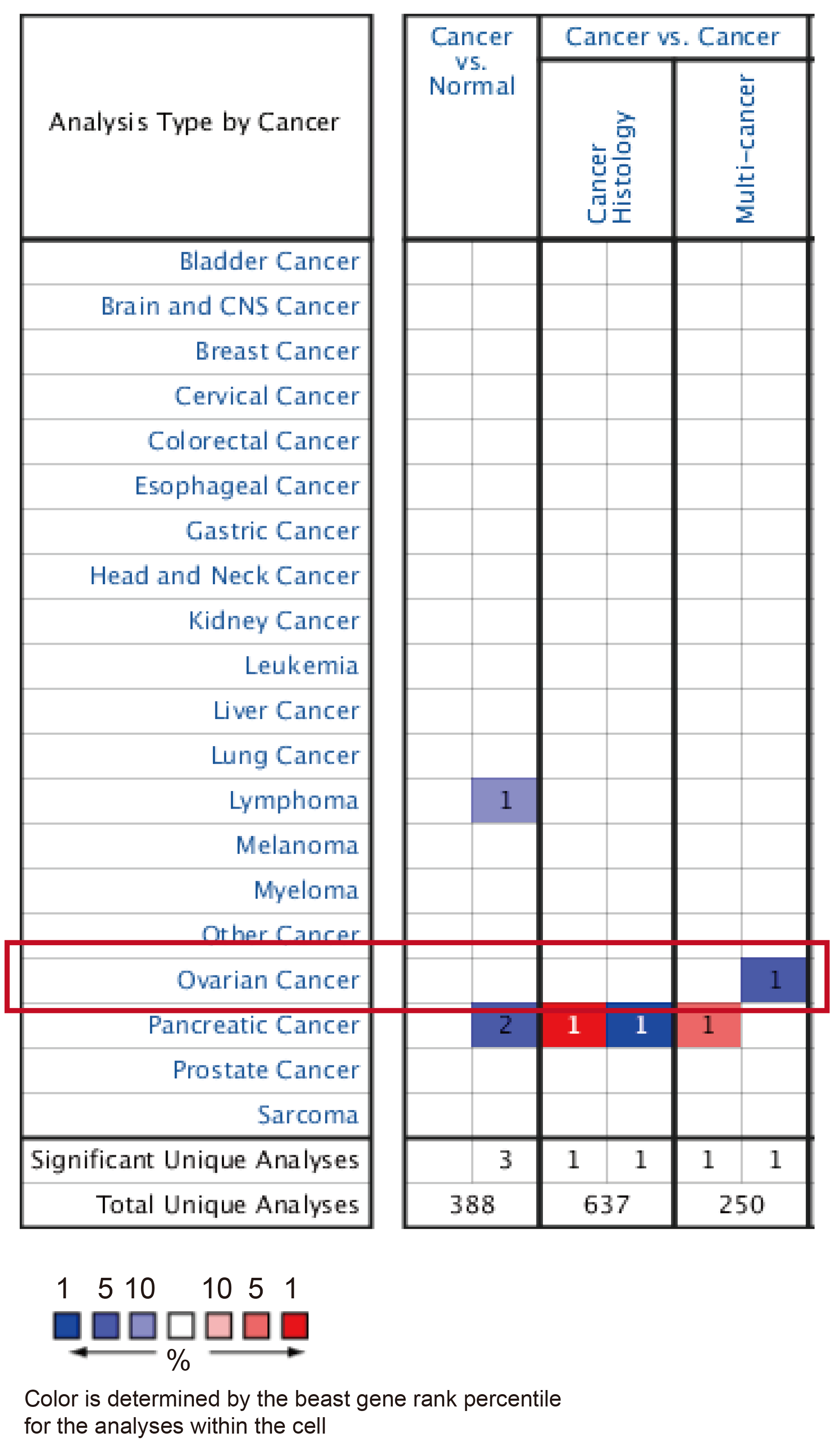

Supplement: Supplementary Figure 2 — Analysis of CTRC expression in different cancers in Oncome database. [file Image_2.TIF]

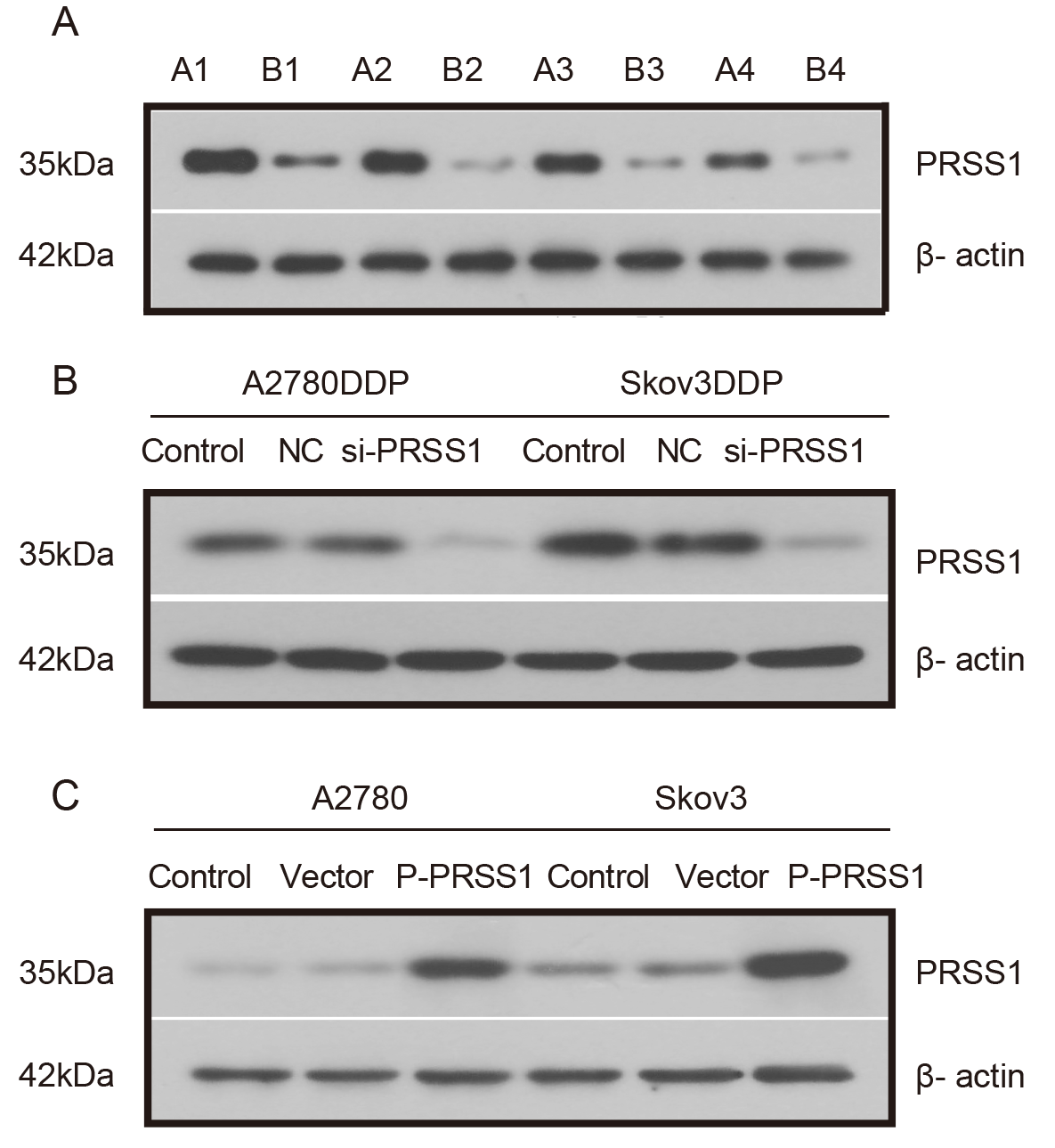

Supplement: Supplementary Figure 3 — Western blot images of PRSS1. PRSS1 was highly expressed in platinum-resistant ovarian cancer tissues (A). Group A, ovarian cancer tissues of platinum-resistant patients; Group B, ovarian cancer tissues of platinum-sensitive patients. Detection of transfection efficiency after the knockdown of PRSS1 in A2780DDP and Skov3DDP cells for 48 h (B). Transfection of A2780 and Skov3 cells with plasmid to overexpress PRSS1; Western blot assay was used to detect PRSS1 transfection efficiency after 48 h (C). All experiments were repeated thrice. [file Image_3.TIF]

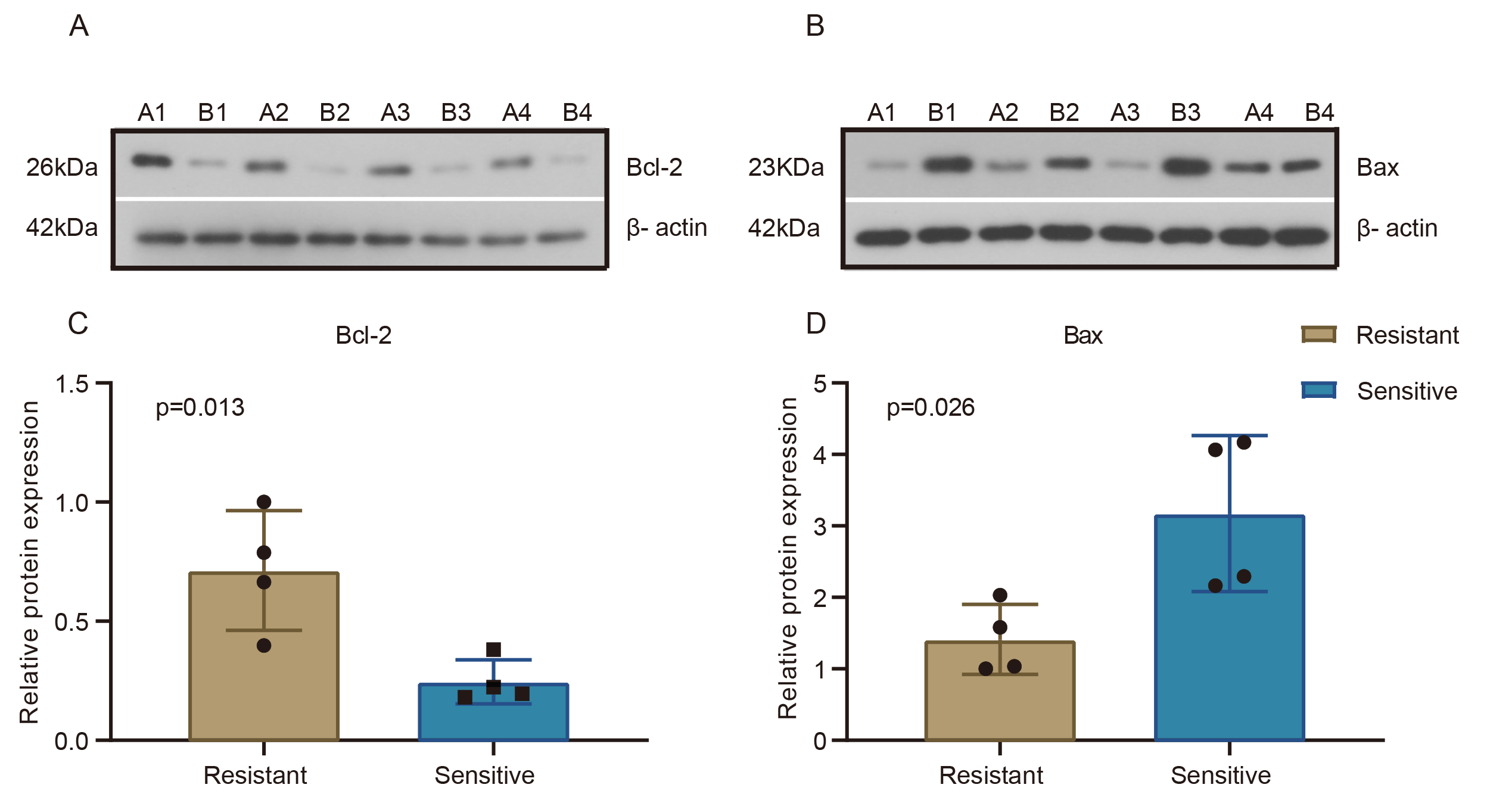

Supplement: Supplementary Figure 4 — Bcl-2 and Bax up-regulated in platinum-resistant and platinum-sensitive tissues, respectively. Western blot assays demonstrated the protein expression of Bcl-2 (A,C) and Bax (B,D) in platinum-resistant/sensitive ovarian cancer tissues. Group A, ovarian cancer tissues of platinum-resistant patients; Group B, ovarian cancer tissues of platinum sensitive patients. The data were presented as the mean ± SD; n = 3. [file Image_4.TIF]

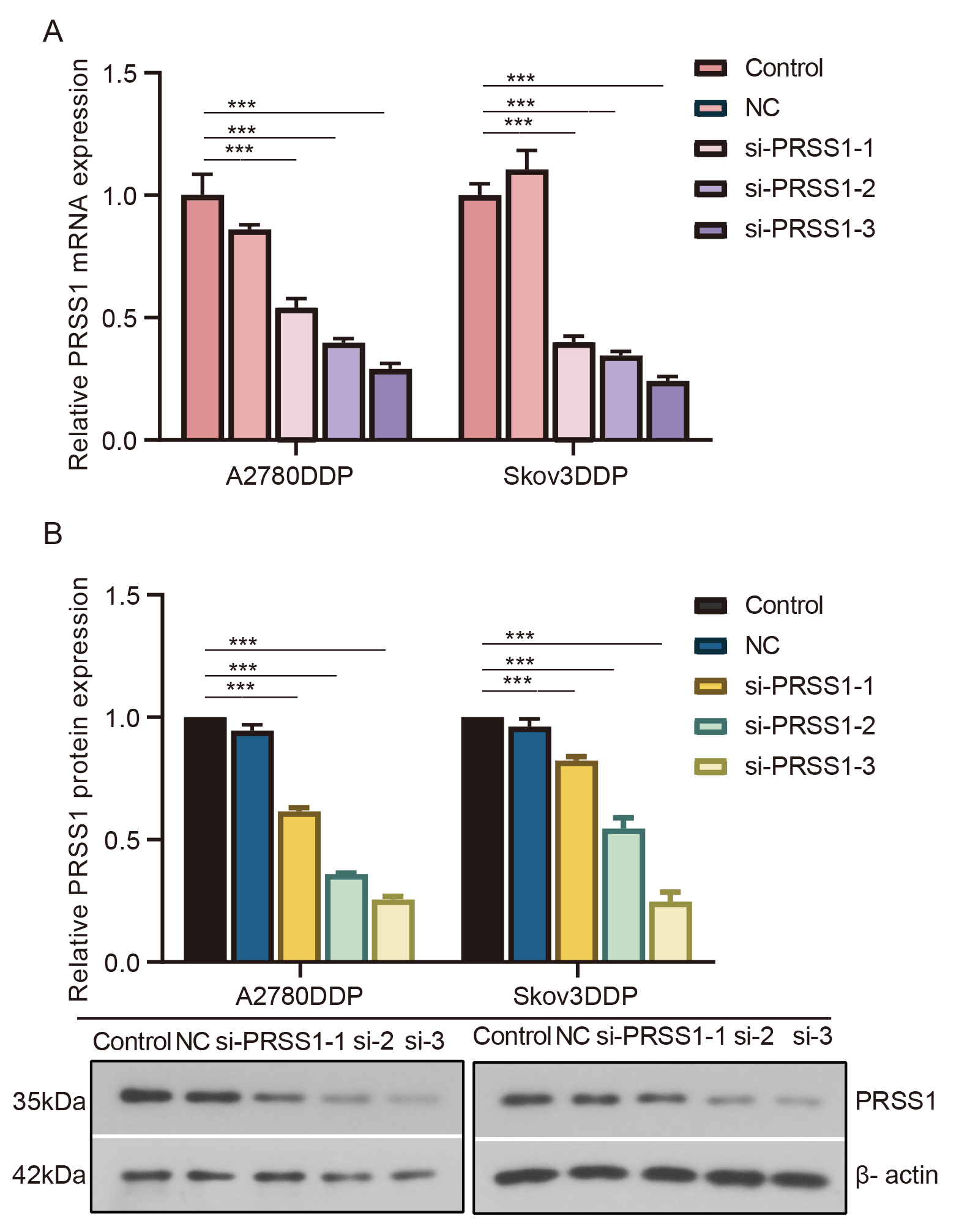

Supplement: Supplementary Figure 5 — Knocked down PRSS1 expression in platinum-resistant cells. RT-qPCR was used to verify the transfection effect of the three Si-PRSS1 24 h after transfection (A). Western blot was applied to detect the transfection effect of the three Si-PRSS1 at 48 h after transfection (B). The data are presented as the mean ± SD; n = 3. ***P < 0.001. [file Image_5.TIF]

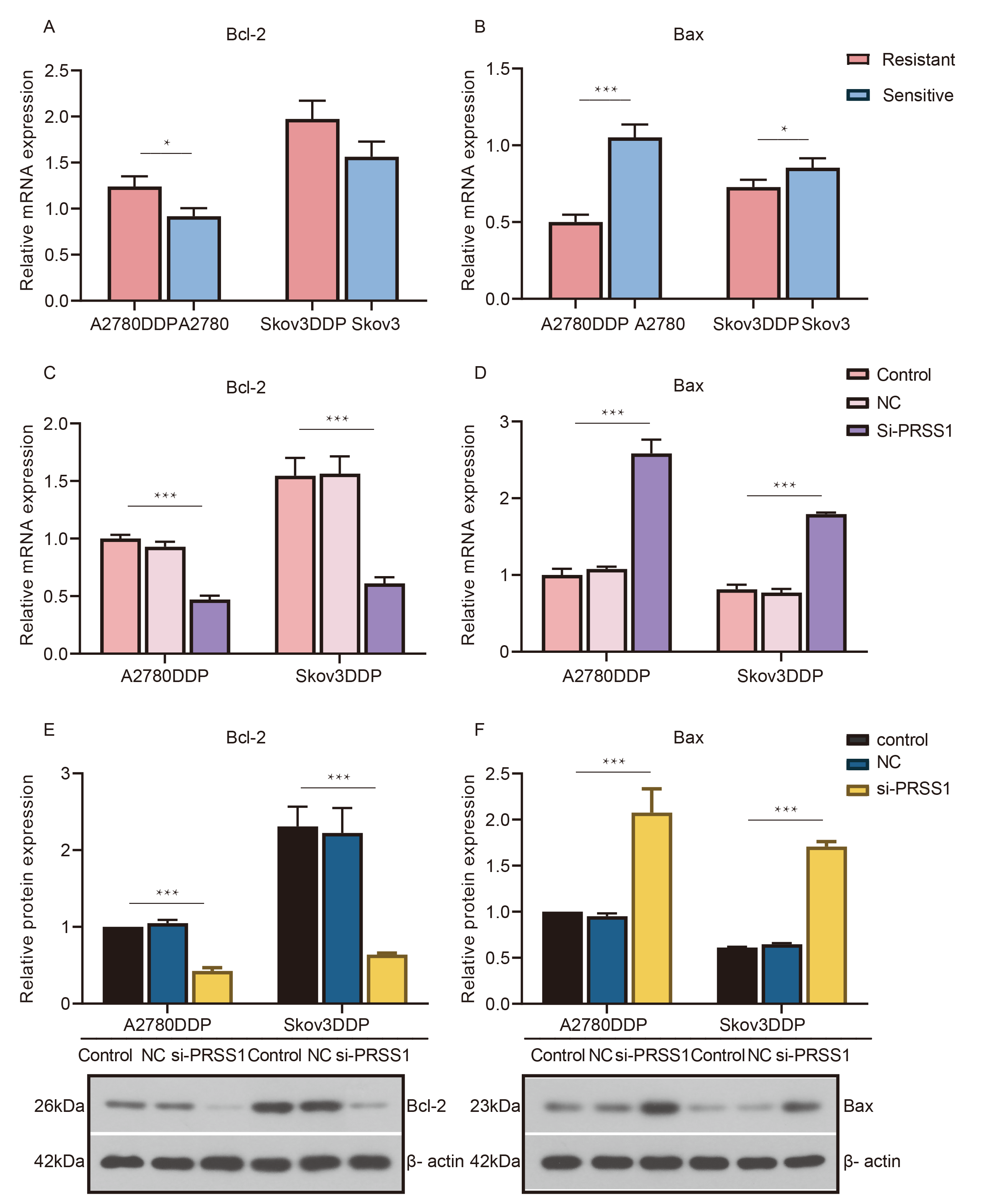

Supplement: Supplementary Figure 6 — Knock down of PRSS1 expression led to an increased ratio of Bax/Bcl-2. Bcl-2 (A) and Bax (B) mRNA expressions of cisplatin-resistant/sensitive ovarian cancer cells were detected by RT-qPCR. Detection of Bcl-2 (C) and Bax (D) mRNA expressions by RT-qPCR assay after knockdown of PRSS1. Western blot assays were used to detect alterations in Bcl-2 (E) and Bax (F) protein expression after PRSS1 downregulation. The data are presented as the mean ± SD; n = 3. *P < 0.05; ***P < 0.001. [file Image_6.TIF]

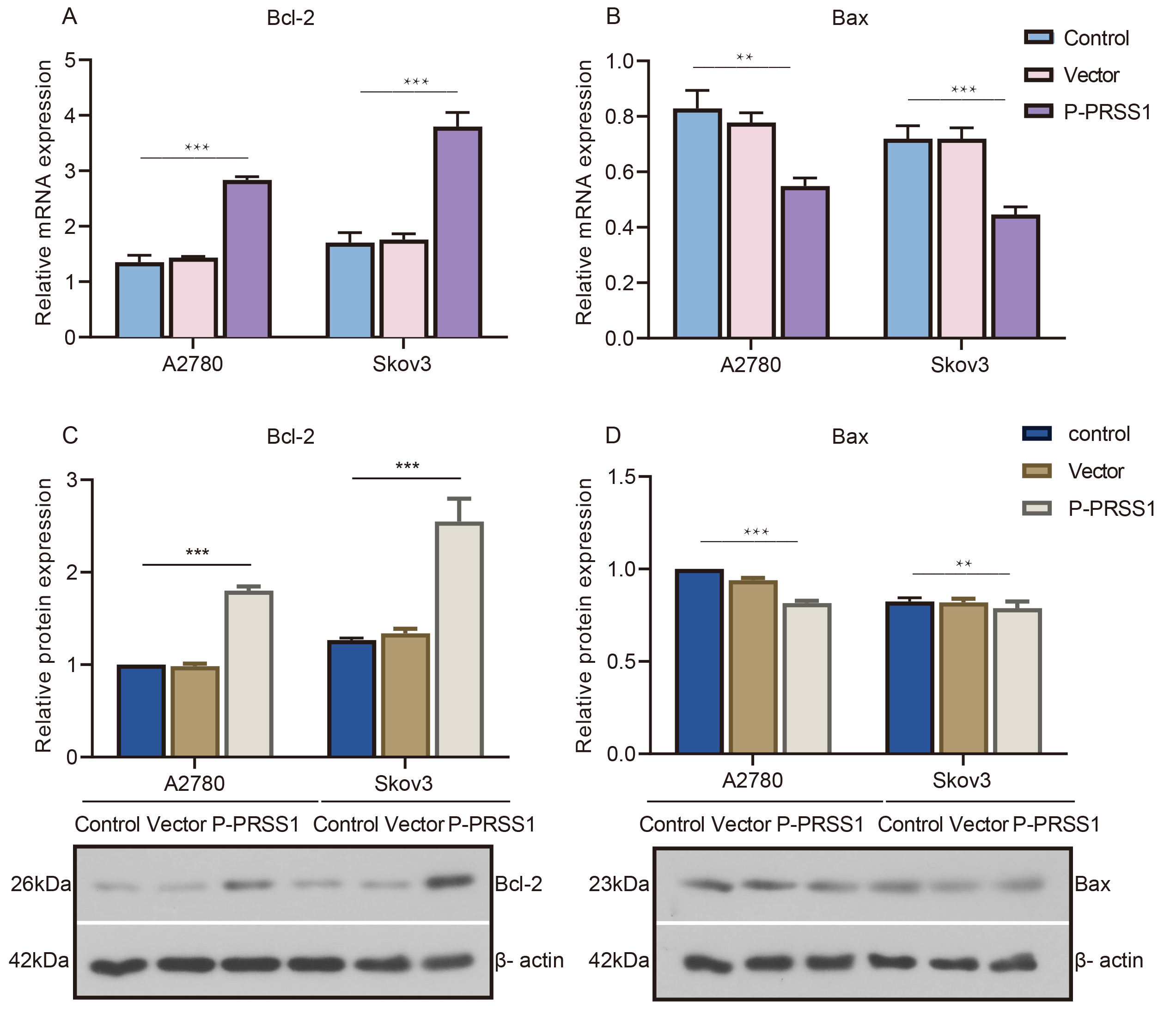

Supplement: Supplementary Figure 7 — Overexpression of PRSS1 decreased the ratio of Bax/Bcl-2. After upregulation of PRSS1 expression, RT-qPCR was used to detect Bcl-2 (A) and Bax (B) mRNA expression levels. Western blot assays were used to detect Bcl-2 (C) and Bax (D) protein expression levels. The data are presented as the mean ± SD; n = 3. **P < 0.01; ***P < 0.001. [file Image_7.TIF]

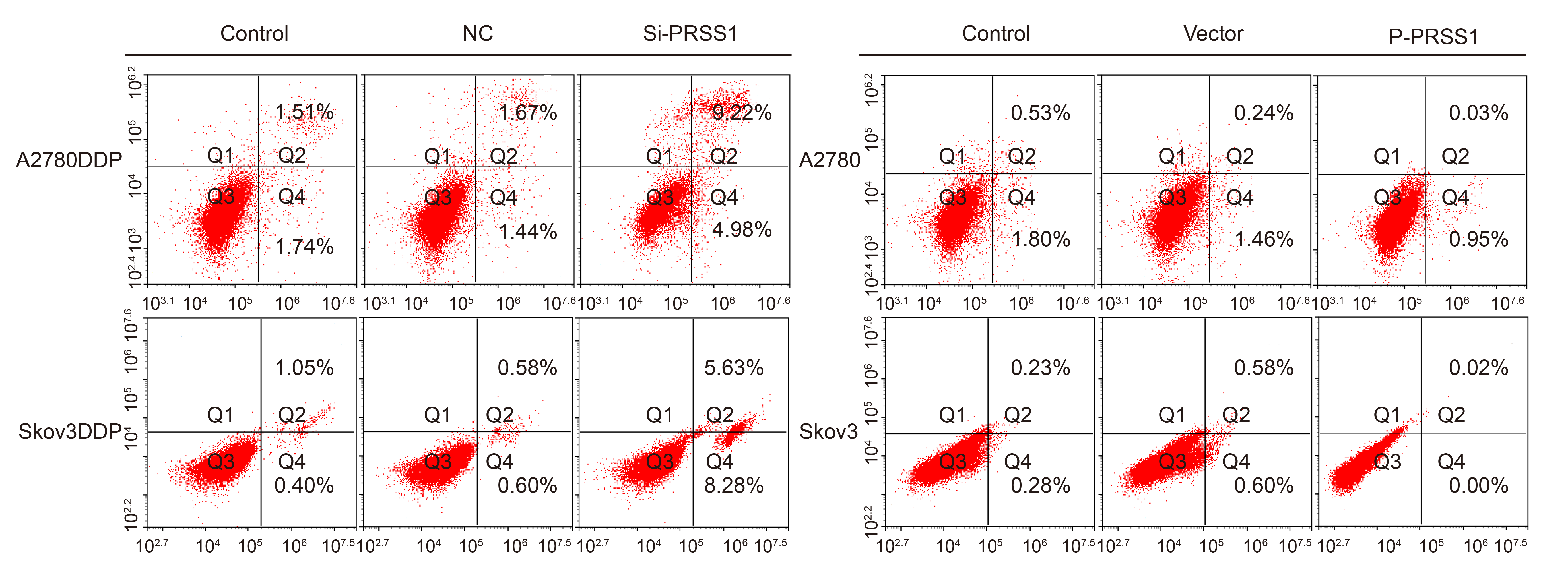

Supplement: Supplementary Figure 8 — Flow cytometry assay of cell apoptosis in ovarian cancer cells. Early apoptotic population in the Q4 gate was characterized with Annexin V (+) and PI (–), and the late apoptotic population in the Q2 gate was characterized with Annexin V (+) and PI (+). All experiments were repeated thrice. [file Image_8.TIF]
