# Supplementary material for: PRSS1 Upregulation Predicts Platinum Resistance in Ovarian Cancer Patients
Source: Front Cell Dev Biol. 2021 Jan 28;8:618341. doi: 10.3389/fcell.2020.618341 (PMC7876278; doi:10.3389/fcell.2020.618341)
Supplement: Supplementary Table 3 — Results of primer sequences. [file Table_3.DOC]

**The sequences of primers**

| **Gene** | **Primer sequence** |
| --- | --- |
| PRSS1 | forward-5’-CGACTACCCAGACGAGC-3’ |
|  | reverse-5’-GCCACCAGAATCACCCT-3’ |
| Bcl-2 | forward-5’-CTGGGAGAACAGGGTACGATAA-3’ |
|  | reverse-5’-GGCTGGGAGGAGAAGATGC-3’ |
| Bax | forward-5’-TTTTGCTTCAGGGTTTCATC-3 |
|  | reverse-5’-GACACTCGCTCAGCTTCTTG-3’ |
| β-actin | forward-5’-GGCACCCAGCACAATGAA-3’ |
|  | reverse-5’-TAGAAGCATTTGCGGTGG-3’ |
| Si-PRSS1 | forward-5’-GCUACAACUGUGAGGAGAATT-3’ |
|  | reverse-5’-UUCUCCUCACAGUUGUAGCTT-3’ |
| Si-PRSS2 | forward-5’-AACGAACAGUGGGUGGUAUTT-3’ |
|  | reverse-5’-AUACCACCCACUGUUCGUUTT-3’ |
| Si-PRSS3 | forward-5’-CCAAUACGACAGGAAGACUTT-3’ |
|  | reverse-5’-CCAAUACGACAGGAAGACUTT-3’ |
