# Supplementary material for: PRSS1 Upregulation Predicts Platinum Resistance in Ovarian Cancer Patients
Source: Front Cell Dev Biol. 2021 Jan 28;8:618341. doi: 10.3389/fcell.2020.618341 (PMC7876278; doi:10.3389/fcell.2020.618341)
Supplement: Supplementary Table 4 — Protein antibodies used for Western blot. [file Table_4.DOC]

**Protein antibodies used for Western blot**

| **Primary Antibody** | **Dilution ratio** | **Second Antibody** | **Dilution ratio** | **Manufacturer** | **Product code** |
| --- | --- | --- | --- | --- | --- |
| **PRSS1 antibody** | 1：1000 | Goat anti-rabbit IgG-HRP | 1：5000 | ABclonal | A1242 |
| **Bax antibody** | 1：500 | Goat anti-rabbit  IgG-HRP | 1：5000 | wanleibio | WL01637 |
| **Bcl-2 antibody** | 1：500 | Goat anti-rabbit  IgG-HRP | 1：5000 | wanleibio | WL01556 |
